# Ectopic gas in the fibular graft after anterior cervical corpectomy and fusion

**DOI:** 10.1186/s12891-021-04874-6

**Published:** 2021-11-29

**Authors:** Satoshi Nozawa, Hiroki Kato, Masaya Kawaguchi, Asae Nozawa, Kazunari Yamada, Chizuo Iwai, Kazunari Fushimi, Kei Miyamoto, Hideo Hosoe, Katsuji Shimizu, Masayuki Matsuo, Haruhiko Akiyama

**Affiliations:** 1 Department of Orthopaedic Surgery, School of Medicine, Gifu University, 1-1 Yanagido, Gifu city, Gifu, 501-1194 Japan; 2 Department of Radiology, School of Medicine, Gifu University, Gifu, Japan; 3 Department of Orthopaedic Surgery, Gifu Prefectural General Medical Center, Gifu, Japan; 4 Department of Orthopaedic Surgery, Gifu Municipal Hospital, Gifu, Japan; 5 Department of Orthopaedic Surgery, Hirano General Hospital, Gifu, Japan

**Keywords:** Ectopic gas, Anterior cervical corpectomy and fusion, Pseudoarthrosis

## Abstract

**Background:**

Ectopic gas in the graft is occasionally encountered upon follow-up computed tomography (CT) after anterior cervical corpectomy and fusion (ACCF). However, most cases lack inflammatory responses and manifestations of infection. Although the clinical significance of ectopic gas in the graft has not yet been established, to the best of our knowledge, no previous studies have described ectopic gas in the graft after ACCF. This study evaluated ectopic gas in the fibular graft upon follow-up CT after ACCF.

**Methods:**

We reviewed 112 patients who underwent ACCF and follow-up CT, with a minimum follow-up period of 3 years. CT images were retrospectively reviewed to confirm the presence of ectopic gas in the graft and bone fusion. Bone fusion was defined as follows: mobility less than 2 mm between spinous processes on the flection-extension radiograph or a bone bridge on CT images.

**Results:**

Of the 112 patients, 30 (27%) patients had ectopic gas in the fibular grafts. Among them, ectopic gas was initially observed 3 months after surgery (early onset) in 23 (77%) patients and 6 months after surgery (late-onset) in the remaining seven (23%) patients. Upon the latest follow-up CT, ectopic gas more frequently remained in late-onset (4/7, 57%) rather than in early-onset (3/23, 13%) cases (*p* = 0.033). Bone fusion was not observed when CT images exhibited ectopic gas in the graft, whereas ectopic gas was not observed when CT images exhibited bone fusion.

**Conclusion:**

Ectopic gas in the fibular graft was observed at both early and late-onset after ACCF; late-onset gas remained significantly. The remaining gas was strongly associated with pseudoarthrosis; therefore, pseudoarthrosis should be considered when ectopic gas in the graft is observed on CT images.

## Introduction

Anterior cervical corpectomy and fusion (ACCF) and strut bone grafting is an established surgical option for the treatment of ossification of the posterior longitudinal ligament (OPLL) and cervical spondylotic myelopathy (CSM). The ilium and fibula strut bones are used in routine surgical procedures to correct (sustain) corpectomy effects. After such procedures, ACCF has been shown to achieve satisfactory clinical outcomes.

Its success in improving pain and disability scores has been well documented [1, 2]. However, the complication of pseudoarthrosis remains a significant challenge, which occurs at a rate of approximately 3–20% in multilevel surgery [3–5]. Kuhns et al. stated that pseudarthrosis after anterior cervical discectomy and fusion (ACDF) has been recognized as a cause of continued cervical pain and unsatisfactory outcomes [5], which can necessitate additional anterior or posterior surgery [4–6].

Gas within the soft tissues usually indicates gas-producing infection caused by anaerobic bacteria or facultative gram-negative bacilli such as *E. coli* and *Klebsiella* spp. Ectopic gas in the graft is occasionally encountered upon follow-up computed tomography (CT) after ACCF, while the majority of cases lack inflammatory responses and manifestations of infection. Although the clinical significance of ectopic gas in the graft has not yet been established, to the best of our knowledge, no previous studies have described ectopic gas in the graft after ACCF. The purpose of this study was to evaluate ectopic gas in the graft upon follow-up CT after ACCF and to determine the clinical significance from the perspective of pseudoarthrosis.

## Materials and methods

This study was approved by the human research committee of the Institutional Review Board, and it complied with the guidelines outlined in the Health Insurance Portability and Accountability Act of 1996. The requirement for written informed consent was waived due to the retrospective nature of the study. We retrospectively reviewed 112 cases (69 males; age range, 21–83 years; mean age, 59.0 years) that underwent ACCF for CSM, OPLL, and cervical disc herniation in our hospital between 2007 and 2018. Patients were included if they had a minimum follow-up period of 3 years. The exclusion criteria included infection, tumor, cases without CT follow-up, and cases requiring reoperation. Bone graft reconstruction was performed using the fibula in 95 patients and the ilium in the remaining 17 patients. The follow-up period after ACCF ranged from 41 to 113 months (mean, 82 months). All patients underwent postoperative CT examinations 3, 6, 12, and 24 months after ACCF. C-reactive protein (CRP) levels, white blood cell (WBC) counts, and erythrocyte sedimentation rates (ESR) were consistently measured during the follow-up period.

### Operative technique

After corpectomy, the defect length was measured, and the fibula or tricortical ilium was harvested. During manual gentle traction of the skull, the bone graft was impacted in the defect. In ACCF with 1- or 2-level corpectomies, anterior cervical plates were indicated. Using a Philadelphia collar, the patients were immobilized until osseous fusion was confirmed. In ACCF with 3-level corpectomies, patients were immobilized with halo vests for 3 months; after that, they wore Philadelphia collars until osseous fusion was obtained.

### Computed tomography imaging

Follow-up CT imaging was performed using an 8-slice CT system (LightSpeed Ultra; GE Healthcare, Milwaukee, WI, USA), a 16-slice CT (LightSpeed Ultra 16; GE Healthcare, Milwaukee, WI, USA), or a 64-slice CT system (Brilliance 64; Philips, Best, The Netherlands). Unenhanced transverse and sagittal multiplanar reconstruction CT images were reconstructed with bone algorithms using a 2.5-mm section thickness and no overlap.

### Imaging assessment

A radiologist with 21 years of post-training experience in musculoskeletal imaging and an orthopedic surgeon with 22 years of post-training experience in spine surgery, individually reviewed all CT images. Any disagreements between the reviewers were resolved by consensus.

First, the reviewers assessed the presence of ectopic gas in the graft upon follow-up CT images. Subsequently, a window level and window width were changed to clearly identify ectopic gas using commercially available DICOM viewers. According to the timing of the initial appearance of ectopic gas, patients with ectopic gas were classified as early-onset (3 months after surgery) and late-onset (6 months after surgery).

Second, the reviewers also assessed the presence of the bone fusion of the graft. Based on the findings from a previous study [7], bone fusion of the graft was defined as follows: mobility less than 2 mm between spinous processes on a flection-extension radiograph or a bone bridge on CT images.

### Statistical analysis

Statistical analysis was performed using SPSS version 22.0 (IBM Corp., Armonk, NY, USA). Fisher’s exact tests were used to compare the amount of the remaining ectopic gas identified in the latest follow-up CT between early- and late-onset cases. *P-*values < 0.05 were considered statistically significant.

## Result

Of the 112 included patients, 30 (27%) patients had ectopic gas in the graft in the follow-up CT images. All 30 bone grafts with ectopic gas were reconstructed using the fibula. Four, 14, 10, and two cases had 1-, 2-, 3-, and 4-level corpectomy, respectively. No signs of infection were found during the follow-up period in any of the patients.

Among 30 patients with ectopic gas, ectopic gas initially appeared 3 months after surgery (early-onset) in 23 patients and 6 months after surgery (late-onset) in the remaining seven patients. Upon the latest follow-up CT, ectopic gas disappeared in 20 (87%) early-onset and three (43%) late-onset cases, whereas ectopic gas remained in three (13%) early-onset and four (57%) late-onset cases. Thus, ectopic gas more frequently remained in the late-onset (4/7, 57%) than in the early-onset (3/23, 13%) cases (*p* = 0.033).

**Fig. 1:**
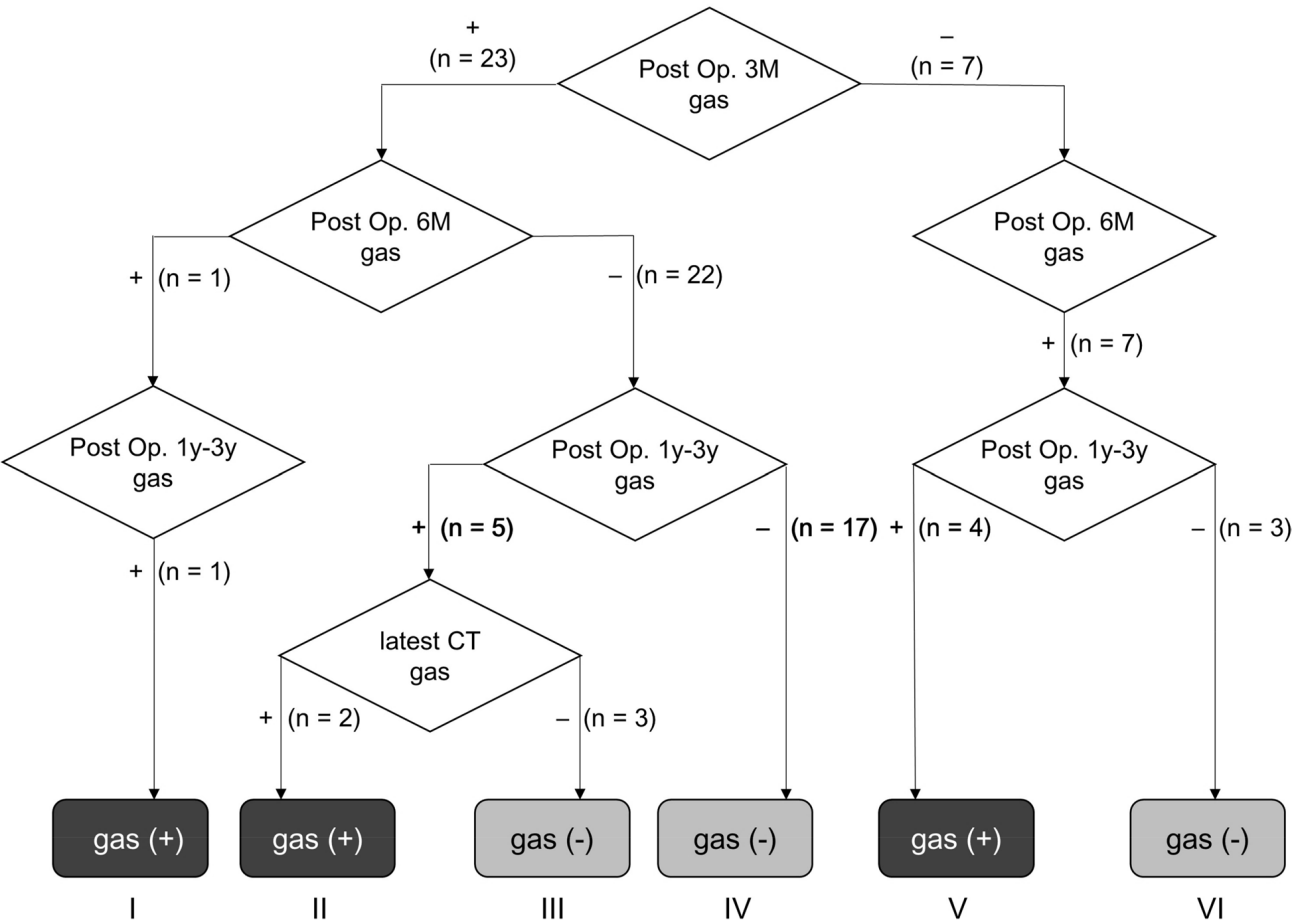
Flowchart of the study population is classified into six groups

**Fig. 2:**
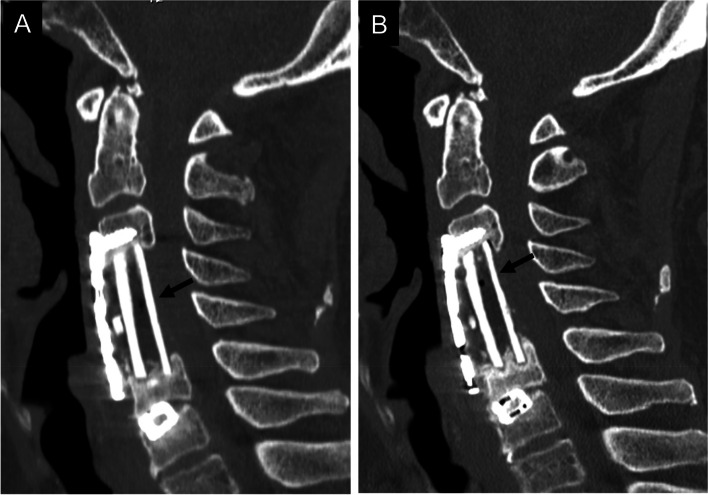
A 72-year-old man underwent anterior cervical corpectomy and fusion due to cervical spondylotic myelopathy (Group I). A two-level corpectomy and a fibula graft were performed. The computed tomography image shows ectopic gas (arrow) in the fibular graft 3 months after surgery (**A**). The gas remained (arrow) without sufficient bone fusion 1 year after surgery (**B**)

**Fig. 3:**
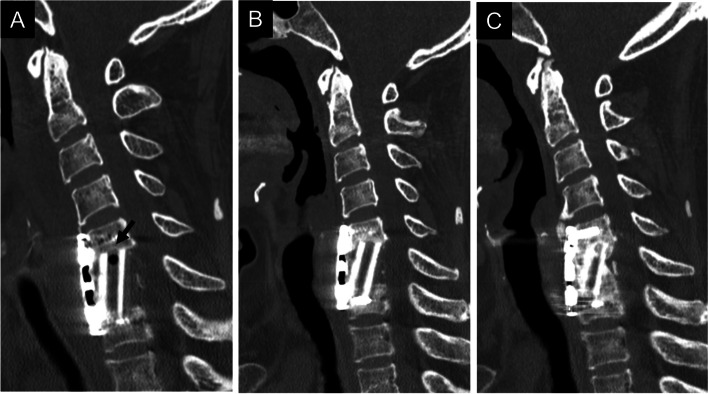
A 54-year-old man underwent anterior cervical corpectomy and fusion due to cervical disc herniation (Group IV). One-level corpectomy and a fibula graft were performed. The computed tomography image showed ectopic gas (arrow) in the fibular graft 3 months after surgery (**A**). The gas disappeared 6 months after surgery (**B**) and 2 years after surgery (**C**) with sufficient bone fusion

**Fig. 4:**
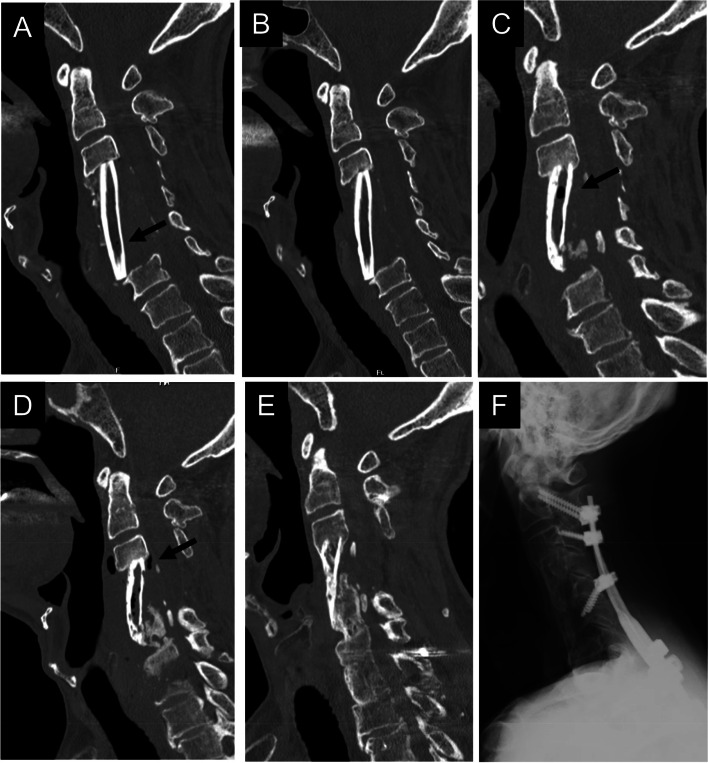
A 65-year-old man underwent anterior cervical corpectomy and fusion due to cervical spondylotic myelopathy (Group II). A three-level corpectomy and a fibula graft were performed. The computed tomography image shows ectopic gas (arrow) in the fibular graft 3 months after surgery (**A**). The gas disappeared 6 months after surgery (**B**), but it appeared again (arrow) with bony erosion of the graft 1 year after surgery (**C**). Three months after graft breakage, the gas (arrow) remained with bony erosion of the graft (**D**). After additional posterior fusion, the gas disappeared as bone fusion was achieved (**E**, **F**)

**Fig. 5:**
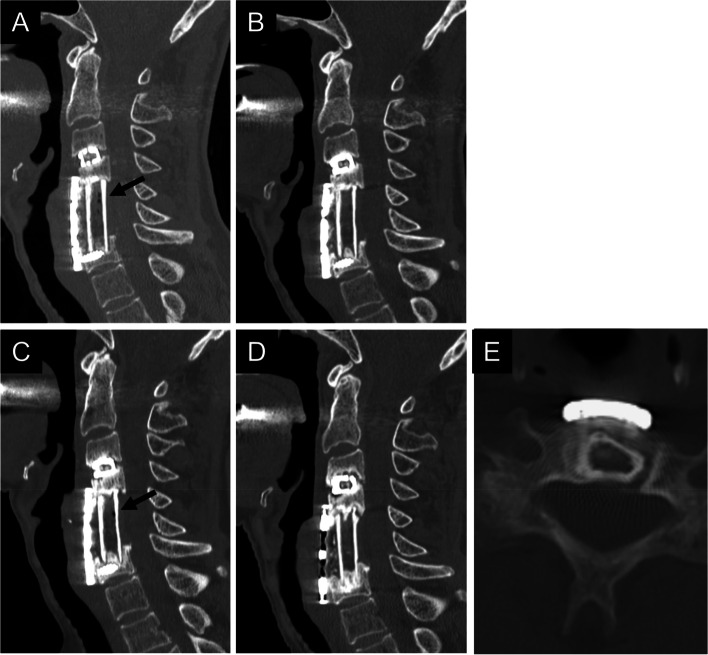
A 71-year-old woman underwent anterior cervical corpectomy and fusion due to cervical spondylotic myelopathy (Group III). A two-level corpectomy and a fibula graft were performed. The computed tomography image showed ectopic gas (arrow) in the fibular graft 3 months after surgery (**A**). The gas disappeared 6 months after surgery (**B**), but it appeared again (arrow) without sufficient bone fusion 1 year after surgery (**C**). The gas disappeared again with sufficient bone fusion 18 months after surgery (**D**, **E**)

**Fig. 6:**
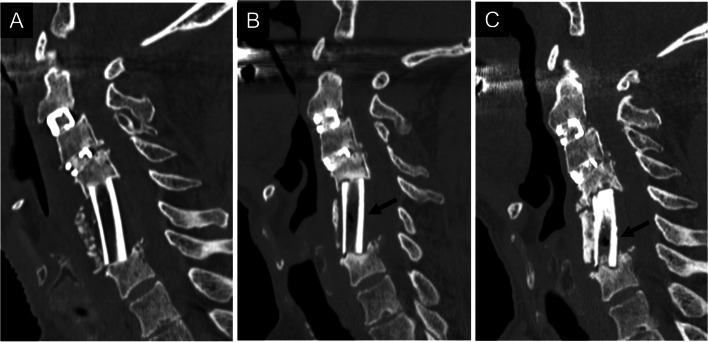
An 82-year-old man underwent anterior cervical corpectomy and fusion due to cervical spondylotic myelopathy (Group V). A two-level corpectomy and a fibula graft were performed. The computed tomography image showed no ectopic gas (arrow) in the fibular graft 3 months after surgery (**A**). The gas appeared 6 months after surgery (**B**) and remained 20 months after surgery (**C**) without sufficient bone fusion

**Fig. 7:**
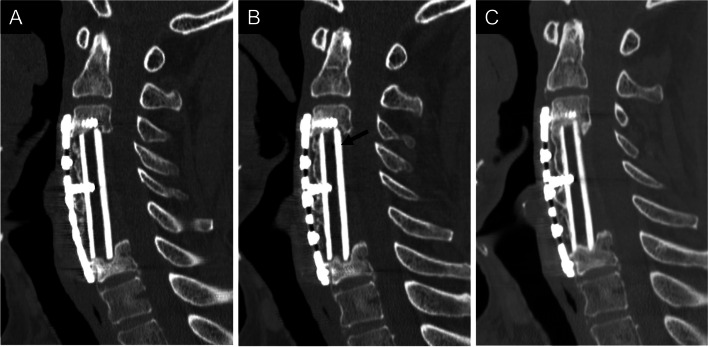
A 70-year-old man underwent anterior cervical corpectomy and fusion due to cervical spondylotic myelopathy (Group VI). A three-level corpectomy and a fibula graft were performed. The computed tomography image showed no ectopic gas (arrow) in the fibular graft 3 months after surgery (**A**). The gas appeared 6 months after surgery without sufficient bone fusion (**B**) but disappeared 2 years after surgery with sufficient bone fusion (**C**)

Thirty cases with ectopic gas were classified into six groups based on the presence or absence of ectopic gas (Fig. 1). Among 23 early-onset cases, ectopic gas continued to appear in one case (Group I, Fig. 2), whereas it disappeared in 22 cases 6 months after surgery. It continued to disappear in 17 cases (Group IV, Fig. 3), while ectopic gas appeared again in five cases 1–3 years after surgery. Among five cases with the reappearance of ectopic gas, ectopic gas continued to remain in two cases (Group II, Fig. 4), whereas it disappeared again in three cases (Group III, Fig. 5) upon the latest follow-up CT images. Conversely, among seven late-onset cases, ectopic gas continued to remain in four cases (Group V, Fig. 6), whereas it disappeared in three cases (Group VI, Fig. 7) 1–3 years after surgery.

The relationship between bone fusion and ectopic gas in the graft upon the latest follow-up CT is summarized in Table 1. Bone fusion was not observed when CT images exhibited ectopic gas in the graft (*n* = 7), whereas ectopic gas was not observed when CT images exhibited bone fusion (*n* = 97).

**Table 1:** Relationship between bone fusion and ectopic gas in the graft on the latest follow-up CT

	Bone fusion + (n = 97)	Bone fusion – (*n* = 15)
Ectopic gas – (*n* = 105)	97	8
Ectopic gas + (n = 7)	0	7

## Discussion

ACCF is an effective surgical procedure for treating CSM, OPLL, and cervical disc herniation [3, 8–13]. The advantage is that the spinal cord is directly decompressed by removing ossified lesions, and the anterior column is stabilized by the strut graft. Although we occasionally encounter ectopic gas in the graft on CT images during the postoperative course, no previous studies have reported ectopic gas in the graft after ACCF. To investigate the clinical significance of this unknown imaging finding, we reviewed 112 cases treated with ACCF. Ectopic gas in the fibular graft was observed at both early and late-onset after ACCF; late-onset gas significantly persisted. The remaining gas was strongly associated with pseudoarthrosis. Pseudarthrosis after ACDF or ACCF has been conclusively proved to be a cause of continued cervical pain and unsatisfactory outcomes [5], which can necessitate additional anterior or posterior surgery [4–6]. Based on the signs of pseudoarthrosis, we could give the patient some advice such as prolonged brace wearing or restriction of neck range of motion. However, it is detrimental to the neck muscle to continue to wear a cervical collar until union is achieved. If the patient has any complaint at the neck, we recommend wearing a soft collar only during exercise. If the complaint remains and impairs activities of daily living, additional salvage posterior fixation using wire or plate is recommended [6].

The presence of ectopic gas in vessels in and around the spine and within the skull usually leads to concerns regarding infections [14]. However, the presence of ectopic gas does not always mean infections. The vacuum disc phenomenon is caused by an accumulation of gas, principally nitrogen, within the crevices of the intervertebral discs or adjacent vertebrae due to disc degeneration [14–16]. Intravertebral gas within a vertebral compression fracture is typically nitrogen, and it is caused by decreased pressure and volume of the vertebra due to the ischemic vertebral collapse [17]. Extra and intradural gas may cause nerve root compression [18, 19]. However, the etiology of ectopic gas in the grafted bone is still unknown.

The accumulation of gas within the bone marrow is observed in various conditions such as osteomyelitis, focal ischemia and osteonecrosis, posttraumatic states, and solitary bone cysts [20]. Another rare condition associated with intraosseous gas includes pneumatocyst, which is a benign cyst-like lesion that is filled with nitrogen within the bone [20–24]. Pneumatocysts most frequently occur in the ilium and the sacrum [24]. The transport of gas from the intervertebral disc into the adjacent subchondral bone via the vertebral endplate may cause intraosseous gas [25]. Laufer et al. speculated that the gas within the pneumatocyst is nitrogen released from the adjacent joints [22].

Coulier et al. speculated the physiological algorithm for the vacuum phenomenon [19, 26]. They hypothesized that in permeable anatomic structures, a sufficiently prolonged distraction allows for progressive penetration of a variable mixture predominantly composed of gas or fluid depending on the nature of the neighboring tissues. When distraction is reduced or compression occurs, gas and/or fluid may be re-injected into the neighboring tissues or forced into blind spaces to form predominantly gaseous or mixed hydro gaseous collections. Repetition of this mechanism results in the “pumping” phenomenon [19].

Jiang et al. systematically reviewed papers on ACCF and summarized that non-union rates were 5.1 and 15.2% for 1-level and 2-level ACCF, respectively. Non-union rates for three disc levels fused were much higher than those for two disc levels fused [27]. Graft dislodgement was found in 20 of the 405 patients treated with ACCF in five studies [8, 28–31].

We believe that the etiology of ectopic gas in the fibular graft after ACCF is similar to that of the vacuum phenomenon or pneumatocyst. Based on our findings we hypothesize, that gas in cases with early onset is caused by migration of air into the graft during surgery, while gas in cases with late onset is caused by micromotion between the graft and the vertebral body. The fact that ectopic gas disappeared after graft fusion was achieved supports our theory.

Moreover, in this series, ectopic gas more frequently remained in cases with late onset (4/7, 57%) than in those with early onset (3/23, 13%) (*p* = 0.033). This suggests that the presence of ectopic gas in the early postoperative stage could predict the gas remaining at postoperative years 1–3, which is related to the non-union of the graft.

## Conclusion

Ectopic gas in the fibular graft was observed at both early and late onset after ACCF; late-onset gas remained significantly. The remaining gas was strongly associated with pseudoarthrosis; therefore, pseudoarthrosis should be considered when ectopic gas in the graft is observed on CT images. The careful observation of ectopic gas in the graft is a simple and useful method for predicting graft fusion.

## Data Availability

The datasets used during the current study are available from the corresponding author on reasonable request.

## References

[1] Bohlman HH, Emery SE, Goodfellow DB, Jones PK (1993). Robinson anterior cervical discectomy and arthrodesis for cervical radiculopathy. Long-term follow-up of one hundred and twenty-two patients. J Bone Joint Surg Am.

[2] Carette S, Fehlings MG (2005). Clinical practice. Cervical radiculopathy. N Engl J Med.

[3] Ikenaga M, Shikata J, Tanaka C (2006). Long-term results over 10 years of anterior corpectomy and fusion for multilevel cervical myelopathy. Spine.

[4] Steinhaus ME, York PJ, Bronheim RS, Yang J, Lovecchio F, Kim HJ (2020). Outcomes of revision surgery for Pseudarthrosis after anterior cervical fusion: case series and systematic review. Global Spine J.

[5] Kuhns CA, Geck MJ, Wang JC, Delamarter RB (2005). An outcomes analysis of the treatment of cervical pseudarthrosis with posterior fusion. Spine (Phila Pa 1976).

[6] Neo M, Fujibayashi S, Yoshida M, Nakamura T (2006). Spinous process plate fixation as a salvage operation for failed anterior cervical fusion. Technical note. J Neurosurg Spine.

[7] Cannada LK, Scherping SC, Yoo JU, Jones PK, Emery SE (2003). Pseudoarthrosis of the cervical spine: a comparison of radiographic diagnostic measures. Spine (Phila Pa 1976).

[8] Emery SE, Bohlman HH, Bolesta MJ, Jones PK (1998). Anterior cervical decompression and arthrodesis for the treatment of cervical spondylotic myelopathy. Two to seventeen-year follow-up. J Bone Joint Surg Am.

[9] Ikenaga M, Shikata J, Tanaka C (2005). Anterior corpectomy and fusion with fibular strut grafts for multilevel cervical myelopathy. J Neurosurg Spine.

[10] Komura S, Miyamoto K, Hosoe H, Fushimi K, Iwai C, Nishimoto H, Shimizu K (2011). Anterior cervical multilevel decompression and fusion using fibular strut as revision surgery for failed cervical laminoplasty. Arch Orthop Trauma Surg.

[11] Komura S, Miyamoto K, Hosoe H, Iinuma N, Shimizu K (2012). Lower incidence of adjacent segment degeneration after anterior cervical fusion found with those fusing C5-6 and C6-7 than those leaving C5-6 or C6-7 as an adjacent level. Clin Spine Surg.

[12] Qin R, Chen X, Zhou P, Li M, Hao J, Zhang F (2018). Anterior cervical corpectomy and fusion versus posterior laminoplasty for the treatment of oppressive myelopathy owing to cervical ossification of posterior longitudinal ligament: a meta-analysis. Eur Spine J.

[13] Nozawa S, Miyamoto K, Sakaguchi Y, Hosoe H, Shimizu K (2004). Ossification of the posterior longitudinal ligament associated with rheumatoid arthritis. Orthopedics.

[14] Sandstrom CK, Osman SF, Linnau KF (2017). Scary gas: intravascular, intracranial, and intraspinal ectopic gas (part III). Emerg Radiol.

[15] Folke Knutsson. The Vacuum Phenomenon in the Intervertebral Discs. Acta Radiologica. 1942;23:173-9. 10.1177/028418514202300207.

[16] Magnusson W (1937). Über die Bedingungen des Hersvortretens der Wirklichen Genlenkspalte auf den Röntgenbild. Acta Radiol.

[17] Maldague BE, Noel HM, Malghem JJ (1978). The intravertebral vacuum cleft: a sign of ischemic vertebral collapse. Radiology.

[18] Ricca GF, Robertson JT, Hines RS (1990). Nerve root compression by herniated intradiscal gas. Case report. J Neurosurg.

[19] Coulier B (2004). The spectrum of vacuum phenomenon and gas in spine. JBR-BTR : organe de la Societe royale belge de radiologie (SRBR) = orgaan van de Koninklijke Belgische Vereniging voor Radiologie (KBVR).

[20] Yamamoto T, Yoshiya S, Kurosaka M, Nagira K, Takabatake M, Hamamoto H, Mineo K (2002). Natural course of an intraosseous pneumatocyst of the cervical spine. AJR Am J Roentgenol.

[21] Hoover JM, Wenger DE, Eckel LJ, Krauss WE (2011). Cervical pneumatocyst. J Neurosurg Spine.

[22] Laufer L, Schulman H, Hertzanu Y (1996). Vertebral pneumatocyst. A case report. Spine (Phila Pa 1976).

[23] Matsukubo Y, Kashiwagi N, Uemura M, Tatsumi S, Takahashi H, Hyodo T, Tomiyama N, Ashikaga R, Ishii K, Murakami T (2013). Intravertebral pneumatocysts of the cervical spine. Neuroradiology.

[24] Hall FM, Turkel D (1989). Case report 526: Intraosseous pneumocyst of the ilium. Skelet Radiol.

[25] Karasick D, Eason MA (1998). Vertebral pneumatocyst mimicking susceptibility artifact on MR imaging. AJR Am J Roentgenol.

[26] Resnick D, Niwayama G, Guerra J, Vint V, Usselman J (1981). Spinal vacuum phenomena: anatomical study and review. Radiology.

[27] Jiang SD, Jiang LS, Dai LY (2012). Anterior cervical discectomy and fusion versus anterior cervical corpectomy and fusion for multilevel cervical spondylosis: a systematic review. Arch Orthop Trauma Surg.

[28] Hilibrand AS, Fye MA, Emery SE, Palumbo MA, Bohlman HH (2002). Increased rate of arthrodesis with strut grafting after multilevel anterior cervical decompression. Spine (Phila Pa 1976).

[29] Nirala AP, Husain M, Vatsal DK (2004). A retrospective study of multiple interbody grafting and long segment strut grafting following multilevel anterior cervical decompression. Br J Neurosurg.

[30] Yonenobu K, Fuji T, Ono K, Okada K, Yamamoto T, Harada N (1985). Choice of surgical treatment for multisegmental cervical spondylotic myelopathy. Spine (Phila Pa 1976).

[31] Uribe JS, Sangala JR, Duckworth EA, Vale FL (2009). Comparison between anterior cervical discectomy fusion and cervical corpectomy fusion using titanium cages for reconstruction: analysis of outcome and long-term follow-up. Eur Spine J.

